# Chitin Nanofibril Application in Tympanic Membrane Scaffolds to Modulate Inflammatory and Immune Response

**DOI:** 10.3390/pharmaceutics13091440

**Published:** 2021-09-10

**Authors:** Serena Danti, Shivesh Anand, Bahareh Azimi, Mario Milazzo, Alessandra Fusco, Claudio Ricci, Lorenzo Zavagna, Stefano Linari, Giovanna Donnarumma, Andrea Lazzeri, Lorenzo Moroni, Carlos Mota, Stefano Berrettini

**Affiliations:** 1 Interuniversity National Consortiums of Materials Science and Technology (INSTM), 50121 Firenze, Italy; bahareh.azimi@ing.unipi.it (B.A.); mario.milazzo@santannapisa.it (M.M.); alessandra.fusco@unicampania.it (A.F.); claudio.ricci@med.unipi.it (C.R.); lorenzo@zavagna.it (L.Z.); giovanna.donnarumma@unicampania.it (G.D.); andrea.lazzeri@unipi.it (A.L.); s.berrettini@med.unipi.it (S.B.); 2 Department of Civil and Industrial Engineering, University of Pisa, 56122 Pisa, Italy; 3 Department of Complex Tissue Regeneration, MERLN Institute for Technology-Inspired Regenerative Medicine, Maastricht University, 6229 ER Maastricht, The Netherlands; s.anand@maastrichtuniversity.nl (S.A.); l.moroni@maastrichtuniversity.nl (L.M.); 4 The BioRobotics Institute, Scuola Superiore Sant’Anna, Viale Rinaldo Piaggio 34, 56025 Pontedera, Italy; 5 Department of Experimental Medicine, University of Campania “Luigi Vanvitelli”, 80138 Naples, Italy; 6 Department of Surgical, Medical, Molecular Pathology and Emergency Medicine, University of Pisa, 56126 Pisa, Italy; 7 Linari Engineering s.r.l., 56121 Pisa, Italy; stefano.linari@linarisrl.com

**Keywords:** nanocomposite, electrospinning, electrospray, polyethylene oxide terephthalate polybutylene terephthalate (PEOT/PBT), eardrum perforation, tympanoplasty, anti-inflammatory, defensin, keratinocytes, shrimp, mushroom, biodegradation

## Abstract

Chitin nanofibrils (CNs) are an emerging bio-based nanomaterial. Due to nanometric size and high crystallinity, CNs lose the allergenic features of chitin and interestingly acquire anti-inflammatory activity. Here we investigate the possible advantageous use of CNs in tympanic membrane (TM) scaffolds, as they are usually implanted inside highly inflamed tissue environment due to underlying infectious pathologies. In this study, the applications of CNs in TM scaffolds were twofold. A nanocomposite was used, consisting of poly(ethylene oxide terephthalate)/poly(butylene terephthalate) (PEOT/PBT) copolymer loaded with CN/polyethylene glycol (PEG) pre-composite at 50/50 (*w/w* %) weight ratio, and electrospun into fiber scaffolds, which were coated by CNs from crustacean or fungal sources via electrospray. The degradation behavior of the scaffolds was investigated during 4 months at 37 °C in an otitis-simulating fluid. In vitro tests were performed using cell types to mimic the eardrum, i.e., human mesenchymal stem cells (hMSCs) for connective, and human dermal keratinocytes (HaCaT cells) for epithelial tissues. HMSCs were able to colonize the scaffolds and produce collagen type I. The inflammatory response of HaCaT cells in contact with the CN-coated scaffolds was investigated, revealing a marked downregulation of the pro-inflammatory cytokines. CN-coated PEOT/PBT/(CN/PEG 50:50) scaffolds showed a significant indirect antimicrobial activity.

## 1. Introduction

Due to its peculiar anatomy and function, the ideal restoration of damaged tympanic membrane (TM) should fulfill several features, which can be critical to enabling an optimal performance, particularly within an infectious and chronically inflamed tissue environment [1]. Along with having to be non-toxic and acousto-mechanically suitable, the biomaterials implanted in the middle ear face the problem of durability, which led to the definition of “middle ear compatibility” as a stringent condition for any prosthetic success [2]. Chronic otitis media (COM) represents a major challenge in eardrum pathology, as it is characterized by a repeatedly infected and long term inflamed middle ear, prone to TM perforation [3,4]. To date, auto/allografts from other tissues are clinically used as patches or replacements to repair the eardrum. However, these have been shown to have suboptimal outcomes [5]. To fill this gap, nanotech manufactures, based on electrospinning and/or 3D printing approaches involving synthetic polymers, have recently been proposed as new routes for TM reconstruction under the tissue engineering paradigm [6,7,8]. By virtue of ultrafine fibers, scaffolds based on biodegradable polymers, like polycaprolactone (PCL) and poly(ethylene oxide terephthalate)/poly(butylene terephthalate) (PEOT/PBT), obtained via electrospinning, have demonstrated an efficient reepithelization in vitro by human TM keratinocytes, as well as colonization by human mesenchymal stem cell (hMSC) and proper collagen synthesis by hMSCs differentiated into fibroblasts [9,10]. These electrospun fibrous meshes do not show any specific features to counteract clinically relevant conditions, such as inflammation or infections. Novel strategies have been proposed incorporating ciprofloxacin-loaded nanoparticles within electrospun PEOT/PBT fibers to fight infections [11]. However, even in case of microbial eradication by drugs, inflammation can self-sustain for a long time in the middle ear, due to inefficient ventilation system and individual susceptibility [2,4]. Highly inflamed tissue environments can in fact slow down the healing process, sustain infection and produce effusions, ultimately resulting in alloplastic device extrusion or accelerated degradation in the ear, without being resolutive for the patients [2,12].

Chitin nanofibrils (CNs) are derived from chitin, available as a waste material from the food industry, by removal of the amorphous components. It has been demonstrated that CNs possess antimicrobial activity as a function of the pH and acetylation degree without hampering hMSC differentiation [13,14]. By virtue of submicrometric size and high crystallinity, CNs lose the allergenic features of chitin and interestingly acquire anti-inflammatory, cicatrizing, and anti-aging activity [15]. In addition, they can be naturally degraded by the body enzymes, such as chitinases and lysozyme; therefore, CNs do not raise strong nanotoxicity concerns [16]. The application of CNs into bioresorbable polymer replacements, such as scaffolds, can follow two main routes: *(i)* as a filler, thus generating a nanocomposite material that slowly releases CNs under biodegradation; and *(ii)* as a surface coating, thus being in subitaneous contact with the tissue microenvironment. The combination of these two routes could ultimately enable a sustained CN availability in a tissue environment for long durations, thus helping to reduce inflammation.

To produce polymeric nanocomposites, CNs must be premixed and efficiently dispersed within a polymer. For their chemistry, CNs can be finely mixed with watery solution soluble biological polymers, such as chitosan [17]. Differently, uniform CN dispersion within hydrophobic polymers, including biodegradable polyesters, like polylactic acid (PLA), in the molten state is difficult to achieve due to the inefficient molecular interactions, which lead to CN segregation and ultimately result in mechanical embrittlement [18]. In the last years, some studies have investigated CN/PLA nanocomposites, pointing out the beneficial role of compatibilizing agents, in particular poly(ethylene glycol) (PEG) and high molecular weight poly(ethylene oxide) (PEO) [19,20,21,22]. According to the premixing methods, CN/PLA nanocomposites have shown ameliorated mechanical properties, as well as improved anti-inflammatory and antimicrobial activity [23,24,25]. Among biodegradable polyesters, PEOT/PBT shows some interesting features, which have proved useful in the medical field, and in particular for middle ear applications [26,27]. Indeed, PEOT/PBT is a family of hydrophilic, semi-crystalline block-copolymers, made by alternating hard and soft segments, which allow mechanical properties to be tuned. In addition, the rate of biodegradation, involving both hydrolysis and oxidation mechanisms affecting PEO segments, is very slow, which can help the material to be stable in an inflamed tissue environment [28]. Therefore, the obtainment of PEOT/PBT nanocomposite incorporating CNs could be suitable for TM replacements by possibly improving the biological properties of the plain copolymer, as CNs would be slowly released upon copolymer biodegradation, and by maintaining optimal mechanical properties, i.e., stiffness, if CNs were finely dispersed inside the PEOT/PBT matrix. In order to have a increased amount of CNs at the surface, coating techniques can be employed. Recently, electrospray has been used for the surface decoration of different polymer fiber meshes with CNs, which ultimately induced an anti-inflammatory behavior of skin keratinocytes [29,30]. Depending on the specific working and environmental parameters used in an electrospinning apparatus nano-to-micrometric fibers and/or particles can be obtained, the latter under the name of electrospray. Such ultrasmall features approach the size sensed by cells, e.g., the extracellular matrix components, thus acting in a biomimetic manner. Unlike bulk alloplastic prostheses applied in tympanoplasty, porous scaffolds provided with suitable surface features can reduce foreign body reactions, such as fibrotic encapsulation, and ultimately promote an appropriate immune response.

In this study, we aimed at investigating the potential applications of CNs in TM scaffolds to modulate the inflammatory and immune response of epithelial cells without compromising their regenerative capability. We utilized a nanocomposite based on PEOT/PBT, using CNs both as a filler and a surface coating for electrospun polymer meshes. A preliminary in vitro degradation study using an otitis simulating fluid was pursued to demonstrate durability of the scaffold in an inflamed environment. We produced nanocomposite fibers via electrospinning, which were further electrosprayed with CNs to have surface coatings. For the latter, we employed CNs obtained from diverse sources, i.e., crustacean, shrimp and mushroom, to assess possible differences in term of pro/anti-inflammatory cytokine expression. Ultimately, cell culture tests were performed by differentiating hMSCs towards TM fibroblasts on the scaffolds to assess cytocompatibility of the nanocomposite, and by using human dermal keratinocytes (HaCaT cells) to investigate the inflammatory and innate immune response. Having an anti-inflammatory, yet durable, TM replacement that also allows TM regeneration, would improve the outcomes in tympanoplasty.

## 2. Materials and Methods

### 2.1. Materials

PEOT/PBT was supplied by PolyVation BV (Groningen, The Netherlands). The copolymer selected and used for this study is the 300PEOT55PBT45. Referring to the commercial nomenclature, the numbers in the name “aPEOTbPBTc” represent: (a) the molecular weight (Mw, g/mol) of PEG and (b, c) the weight ratios of the PEOT and PBT blocks, respectively. CNs (2% *w/w*) (from crustaceans) dispersion in water was supplied by Texol s.r.l., (Alanno, Italy); shrimp-based chitin nanofibrils (sCN) (1.5% *w/w* in water) and mushroom-based chitin nanofibrils (mCN) (1.5% *w/w* in water) were supplied by Celabor s.c.r.l. (Brussels, Belgium). PEG with a number average molecular weight (Mn) of 4000 g/mol (code: 81240) was supplied by Sigma Aldrich (Milan, Italy) in the form of platelets, chloroform by Merck KGaA (Darmstadt, Germany), and hexafluoro-2-propanol (HFIP) of analytical grade by Biosolve BV (Valkenswaard, The Netherlands). Lyophilized bovine serum albumin (BSA) was supplied by BIOWEST S.A.S. (Nuaillé, France). Lysozyme (20,000 UI/g) was supplied by Eurobio Scientific (les Ulis, France). Absolute ethanol (EtOH), phosphate buffered saline (PBS), NaCl salt, fluconazole, penicillin and streptomycin (pen-strep), gelatin (type B, from bovine skin), and MgCl_2_ were supplied by Sigma-Aldrich via Merck KGaA (Darmstadt, Germany), sterile saline solution and sterile 5% glucose saline solution by Fresenius Kabi (Bad Homburg vor der Höhe, Germany), formalin by Bio-Optica (Milan, Italy). Heat-inactivated FBS and 4′,6-diamidino-2-phenylindole (DAPI) were obtained by Invitrogen via Thermo Fisher Scientific (Waltham, MA, USA). StemMACS ChondroDiff medium was obtained from Miltenyi Biotec (Bergisch Gladbach, Germany). Human mesenchymal stromal cells (hMSCs) were supplied by Merck Millipore S.A.S. (Burlington, MA, USA). AlamarBlue was bought from Thermo Fisher Scientific (Waltham, MA, USA). Immortalized human keratinocytes, HaCaT cell line, were commercially obtained from ATCC-LGC Standards (Milan, Italy). LC Fast Start DNA Master SYBR Green kit and TRizol were obtained from Roche Applied Science (Euroclone S.p.A., Pero, Italy). Phalloidin i-Fluor 488, Anti-collagen type I were bought from AbCam (Cambridge, MA, USA). Secondary antibody was obtained by Cell Signalling Technology (Danvers, MA, USA).

### 2.2. Nanocomposite Preparation

To obtain uniform PEOT/PBT/(CN/PEG) composites, melt blending was used. PEG was added to homogeneously dispersed CNs in PEOT/PBT via pre-composite preparation, starting from an aqueous CN dispersion of 2% *w/w*. The use of PEG as a dispersing agent has been previously reported to effectively avoid CN aggregation in the melt during extrusion [24]. Pre-composites of CN/PEG at 50:50 wight ratios were employed. PEG was added to the aqueous CN and the solution was left under magnetic stirring for 2 h, at room temperature (RT), to obtain a uniform CN/PEG blend. The solution was transferred to a Petri dish and left drying overnight under ventilated hood until solidification. The CN/PEG pre-composite was dispersed in the PEOT/PBT melt during extrusion to obtain a final concentration of 2% CNs by weight in the copolymer. The CN/PEG pre-composite was homogenized using mortar and pestle and fed together with the PEOT/PBT pellets into a Thermo Haake Minilab II twin screw extruder (Haake, Vreden, Germany) at 165 °C. PEOT/PBT nanocomposite filament was finally obtained.

### 2.3. Production of TM Scaffolds 

Electrospinning was performed with a Fluidnatek LE−100 apparatus (Bioinicia S.L., Spain) to fabricate a homogeneous mesh of nanofibers. PEOT/PBT composite filament was palletized and dissolved using a concentration of 18% *w/v* in a mixture of chloroform and hexafluoro−2−propanol 70:30 (*v/v*) by stirring overnight at RT [10]. Optimization of the processing parameters was performed on a rotating drum collector covered with aluminum foil, and led to the conditions reported in Table 1. Thin samples were produced to replicate an optimal TM replacement. Following the method reported in a previous study [8], the samples were collected over a polypropylene (PP) sheet with holes (1 cm diameter) to achieve a representative mesh surface for cell cultures, facilitate the removal of the mesh from the substrate, and have the thicker border required to manipulate the thin and flexible samples. 

To achieve a full functionalization of the surface of the fibers, the possibility of directly electrospraying CNs over the scaffolds was investigated. CNs from different providers and sources were dissolved at 0.52 w % in aqueous acetic acid and distilled water (50:50 *w/w*). The solution was magnetically stirred for 3 h until uniform appearance. Electrospray was run for 20 min using an electrospinning apparatus (Linari Engineering s.r.l., Pisa, Italy). The collector for parameter optimization consisted of a flat sheet of grounded aluminum foil. A distance of 10 cm from the needle tip, flow rate of 0.298 mL/h, and a voltage of 15 kV were employed for CNs (from Texol). A flow rate of 0.136 mL/h, a voltage of 17 kV, and a distance of 7 cm were employed for sCN and mCN (from Celabor). To decorate the fiber surface, the electrospun PEOT/PBT/ (CN/PEG 50:50) meshes were fixed over the aluminum foil, and same electrospray parameters were employed.

### 2.4. TM Scaffold Characterization

Morphological analysis of the samples was performed using field emission electron scanning microscopy (SEM) with FEI FEG-Quanta 450 instrument (Field Electron and Ion Company, Hillsboro, OR, USA) and Inverted optical microscope (Nikon Ti, Nikon Instruments, Amsterdam, The Netherlands). The samples were sputtered with platinum for analysis. Image J software (version 1.52t, nih.gov) was used to evaluate the size (i.e., the longest dimension) of the electrosprayed CNs. The average and standard deviation of 50 measurements was reported for each sample. Fourier Transform Infrared Spectroscopy (FTIR) using Nicolet T380 apparatus (Thermo Scientific, Waltham, MA, USA), equipped with a Smart ITX ATR attachment with a diamond plate, was used for chemical structure characterization of the samples to check the presence of CNs.

### 2.5. Degradation Study in Otitis-Simulating Conditions

For the in vitro degradation study, a fresh solution was prepared every week 1 h in advance. All the components were dissolved in distilled water to reach concentrations of 0.338 mg/mL of glucose, 6.27 mg/mL of NaCl, 54 mg/L of albumin and 2 mg/mL of lysozyme. The pH of the solution ranged in 7-8, consistent with the values reported in literature [31]. Samples of pristine PEOT/PBT and nanocomposite fibers (n = 4) were cut out of the collected mesh by keeping a thin PP border to handle the samples and each of them was further placed in 3 mL degrading solution. Once a week, the samples were removed from the bath, washed twice in 10 mL distilled water, placed in 2 mL of absolute EtOH for 5 min together with the flasks (to avoid microbial contamination), let dry under ventilated hood and re-placed in the degradation bath at 37 °C. Every month, one sample for each type was taken, washed for ≥20 min in 10 mL water and 20 min in 10 mL absolute EtOH at RT, let dry under ventilated hood and then analyzed via SEM imaging. The samples were kept in the degradation bath for 4 months.

### 2.6. Cytocompatibility Evaluation

The scaffolds, cut out with 1 mm PP border to avoid crumpling, were sterilized with absolute EtOH overnight, followed by three rinses in 3× fluconazole and pen-strep PBS solution for 10 min. Subsequently, the materials were stored in a 24-well plate. HMSCs were used to investigate the interaction of the nanocomposite with connective tissue [9]. Briefly, the cells, defrosted and expanded as from the provider’s instructions, were seeded at 2 × 10^5^ onto the scaffolds (i.e., pristine and nanocomposite PEOT/PBT), pretreated with sterile filtered 2% (*w/v*) gelatin/water solution for 30 min, in triplicate and cultured for 1 week in humidified incubator under standard conditions (i.e., 37 °C, 5% CO_2_) using Chondrodiff complete medium at 1 mL/sample, to be replaced every 3 days. AlamarBlue^®^ test was performed on days 1, 4, and 8 to monitor cell metabolic activity, as from the manufacturer’s instructions. At the end of the culture, the nanocomposites were fixed in 1% (*w/v*) neutral buffered formalin for 10 min at 4 °C. Finally, the specimens were dehydrated in 70% (*v/v* %) EtOH/water solutions for 30 min and dried in vacuum oven at 37 °C overnight, then processed for SEM analysis. For fluorescence staining, the fixed samples were permeabilized in 0.1% (*v/v*) Triton X-100 in PBS 1× for 5 min, incubated with phalloidin for 90 min RT in the dark, following manufacturer’s instructions and finally with 10 µg/mL DAPI in PBS 1× for 10 min in the dark. After each steps, washings in PBS 1× were performed. For immunofluorescence, after fixation, the samples were permeabilized in 0.2% Triton X-100 in PBS 1× for 10 min and incubated with primary antibody anti-collagen type I (ab34710) 1:500, diluted in 0.1% (*w/v*) BSA/PBS 1× in humidified chamber overnight at 4 °C. The following day, the samples were incubated with anti-mouse and anti-rabbit secondary antibody conjugated with TRITC fluorophore diluted 1:70 in PBS 1×, for 1 h at RT in the dark. Finally, the specimens were incubated with 10 µg/mL DAPI in PBS 1× for 10 min in the dark. After each step, washing in PBS 1× was performed. The outcomes of fluorescence staining and immunostaining were observed with an inverted microscope equipped with fluorescence and with a digital camera (Nikon Eclipse TI, Nikon Instruments, Amsterdam, The Netherlands).

### 2.7. Immune and Inflammatory Response Evaluation

Immortalized human dermal keratinocytes, HaCaT cells, were used as a model of TM epithelial cells to investigate the immune and inflammatory response due to the interaction with CNs. They were cultured in DMEM supplemented with 1% pen-strep, 1% L-glutamine, and 10% fetal calf serum at 37 °C in air and 5% CO_2_. The HaCaT cells were seeded in 12-well plates until 80% of confluence and incubated for 24 h with the films. The samples, sterilized overnight in EtOH and rinsed three times with PBS, were placed on the bottom of 6-well plates, then HaCaT cells were plated on them and incubated for 6 h and 24 h. At the end of the experiment, the mRNA was extracted from the cells and the levels of expression of the proinflammatory cytokines: interleukin (IL)-1β, IL-1α, IL-6, IL-8, and tumor necrosis factor (TNF)-α, the anti-inflammatory cytokine transforming growth factor (TGF)-β, and antimicrobial peptide human beta defensin (HBD)-2 were evaluated by real-time reverse transcriptase polymer chain reaction (RT-PCR). Briefly, the total RNA was isolated with TRizol, and 1 µm of RNA was reverse-transcribed into complementary DNA (cDNA) using random hexamer primers at 42 °C for 45 min, according to the manufacturer’s instructions. PCR was carried out with the LC Fast Start DNA Master SYBR Green kit using 2 µL of cDNA, corresponding to 10 ng of total RNA in a 20 µL final volume, 3 mM MgCl_2_, and 0.5 µM sense and antisense primers (Table 2). The results were normalized by the expression of the same cytokine in untreated cells, as a control (ctrl).

### 2.8. Statistical Analysis

One-way ANOVA was applied to evaluate AlamarBlue assay; mRNA expression toward untreated cells (controls; ctrl) was assessed using Student’s *t*-test. Probability (*p*) values < 0.05 were considered as statistically significant differences.

## 3. Results

### 3.1. TM Scaffold Characterization

**Figure 1:**
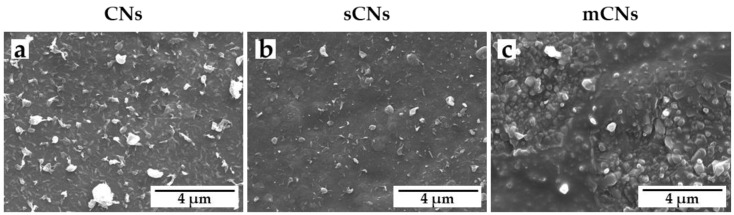
SEM micrographs of electrosprayed CNs from different sources: (**a**) CNs (from crustaceans by Texol s.r.l.); (**b**) sCNs (from shrimps, by Celabor s.c.r.l.); and (**c**) mCNs (from mushroom by Celabor s.c.r.l.). Scale bar: 4 μm, high voltage (HV) 10 kV, magnification 30,000×.

**Figure 2:**
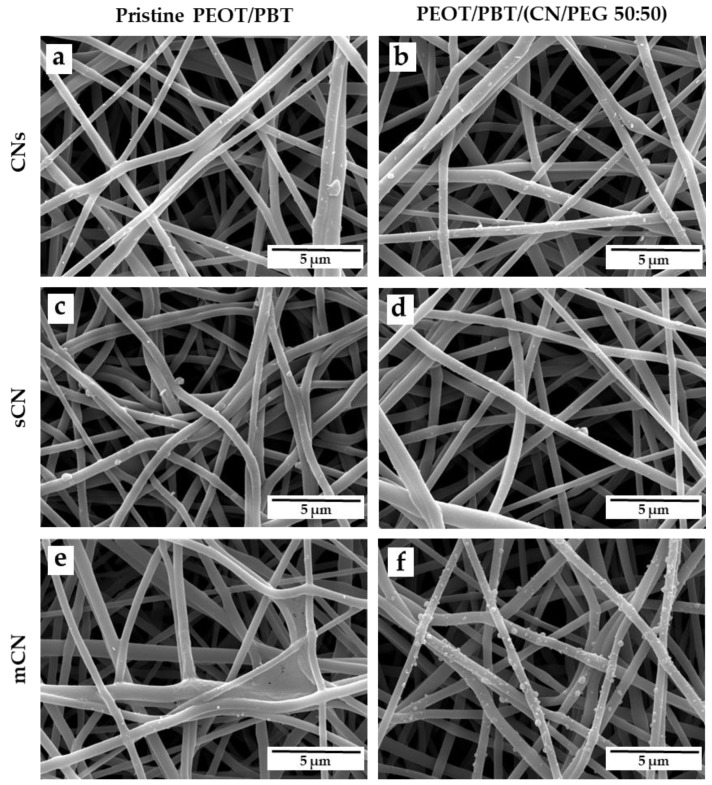
SEM micrographs of electrosprayed CNs from different sources, namely CNs (from crustaceans by Texol s.r.l.), sCNs (from shrimps, by Celabor s.c.r.l.), and mCNs (from mushroom by Celabor s.c.r.l.) on (**a**,**c**,**e**) PEOT/PBT scaffolds; and (**b**,**d**,**f**) PEOT/PBT/ (CN/PEG 50:50) scaffolds. Scale bar: 5 μm, high voltage (HV) 10 kV, magnification 20,000×.

Suspensions of different CN sources were electrosprayed using aluminum foil (Figure 1), as well as the pristine copolymer and the nanocomposite fiber scaffolds (Figure 2) as collecting substrates.

On aluminum, CNs were well dispersed, displayed uniform surface decoration, and showed an average size of 0.16 ± 0.13 µm for CNs (Figure 1a), 0.27 ± 0.09 µm for sCN (Figure 1b), and 0.24 ± 0.09 µm for mCN (Figure 1c). To functionalize the TM scaffolds, both the pristine PEOT/PBT copolymer and the nanocomposite were electrospun into fibrous meshes with submicrometric size using identical operating conditions. Figure 2 shows SEM analysis of PEOT/PBT and PEOT/PBT/ (CN/PEG 50:50) electrospun scaffolds, decorated with CNs from different sources via electrospray. Additionally, using the PEOT/PBT-based electrospun meshes as substrates, CNs with uniform size and morphology were homogeneously detected on the surface of the fibers in all the samples. Furthermore, electrosprayed mCN displayed a better adhesion to the nanocomposite fiber surface (Figure 2f), with respect to CNs extracted from fish sources.

**Figure 3:**
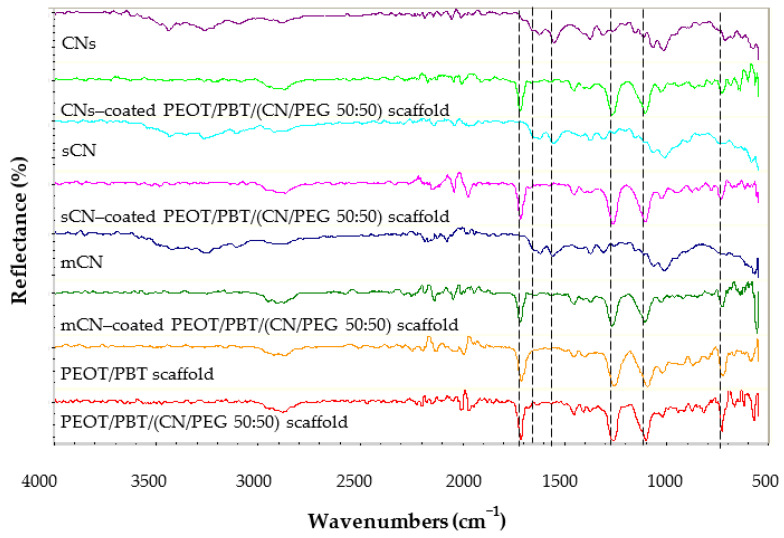
Fourier-transform infrared spectroscopy (FTIR) spectra of pristine CNs from different sources, namely CNs (from crustaceans by Texol s.r.l.), sCNs (from shrimps, by Celabor s.c.r.l.), and mCNs (from mushroom by Celabor s.c.r.l.) in powder form, and PEOT/PBT or PEOT/PBT/ (CN/PEG) scaffold surface coated with electrosprayed CNs from different sources.

FTIR analysis was performed over all the samples to check for CN presence and possible changes in the chemical composition following the different manufacturing processes (Figure 3). The dried pristine CNs were characterized by FTIR in ATR mode. The characteristic bands of CN are 1010 cm^−1^ and 1070 cm^−1^, typical of C–O stretching, 1552 cm^−1^ attributed to amide II, 1619 cm^−1^ and 1656 cm^−1^ attributed to amide I, 2874 cm^−1^ attributed to C–H stretching, 3102 cm^−1^ and 3256 cm^−1^ attributed to N–H stretching of the amide and amine groups, and 3439 cm^−1^ attributable to O–H stretching.

Figure 3 shows the FTIR spectra of pristine CNs from different sources, PEOT/PBT electrospun scaffolds, PEOT/PBT/(CN/PEG 50:50) electrospun scaffolds and the scaffolds functionalized with different types of electrosprayed CN. The main characteristic bands of CN were observed on the PEOT/PBT/(CN/PEG 50:50) scaffolds functionalized with electrosprayed CNs from different sources. These observations confirmed the presence of CNs on the scaffold surfaces. The main characteristic bands of PEOT/PBT can be also observed on the surface of scaffolds coated with electrosprayed CNs. The obtained results demonstrated that the solvent system did not affect CN structure.

### 3.2. Degradation in Otitis-Simulating Solution

**Figure 4:**
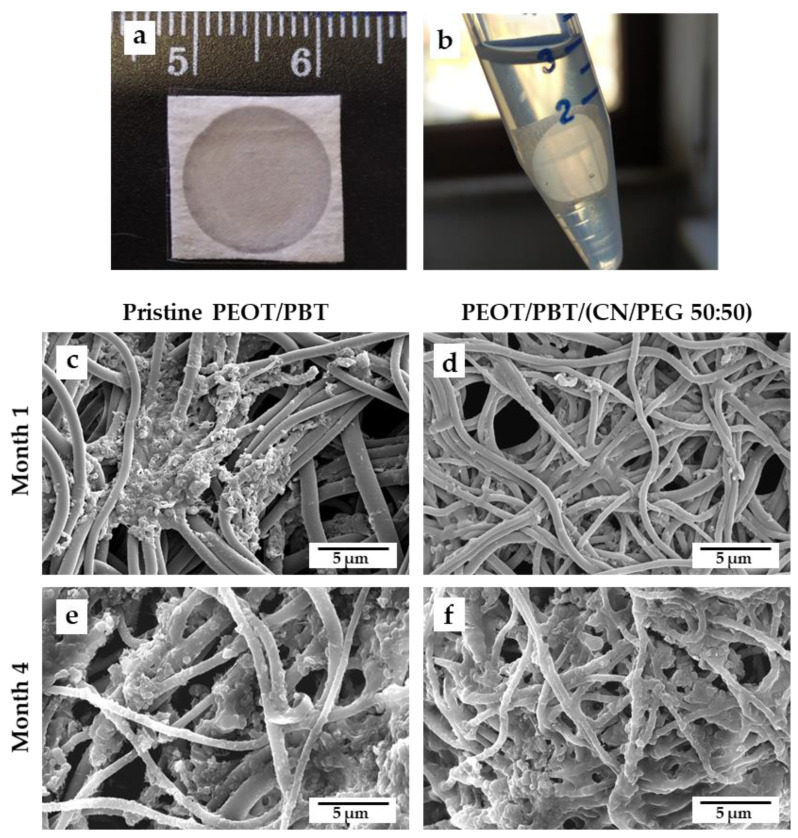
Degradation test of PEOT/PBT and PEOT/PBT/(CN/PEG 50:50) in an otitis-simulating fluid: (**a**,**b**) real size TM scaffold placed in the fluid at month 0; (**c**–**f**) SEM micrographs showing the fiber morphology of (**c**,**e**) PEOT/PBT, and (**d**,**f**) PEOT/PBT/(CN/PEG 50:50) meshes after (**c**,**d**) 1 month, and (**e**,**f**) 4 month incubation in the fluid. Scale bar: 5 μm, high voltage (HV) 10 kV, magnification 16,000×.

To assay the durability of these TM scaffolds, in real size, within an inflamed middle ear environment, the PEOT/PBT and PEOT/PBT/(CN/PEG 50:50) were incubated for 4 months in an otitis-simulating solution at 37 °C (Figure 4a,b).

Every month, the samples were observed via SEM, which revealed them to be covered by a protein layer imputable to albumin (as confirmed via FTIR spectra, data not shown). The samples were thus washed to remove the protein layer and observe the fiber structure. Due to the protein deposition and to the light weight of the thin samples, weight measurements to assess weight loss upon degradation were not reliable. Figure 4c–f shows SEM analysis of fiber surfaces performed after 1 month and 4 month degradation, both for the PEOT/PBT and PEOT/PBT/(CN/PEG 50:50) scaffolds. An organic coating was well visible in all the samples. After 2 months of degradation, no sign of erosion was present on the surface of the fibers. The high presence of material retained during the degradation test made direct investigation of the fiber surfaces very difficult for the last two months, but in the cleanest zones of the meshes reported in Figure 4c–f, no sign of surface erosion or break was present. These results confirm the durability of these copolymeric fibers, including the nanocomposite, in a harsh body environment.

### 3.3. Interaction of TM Scaffolds with hMSCs

**Figure 5:**
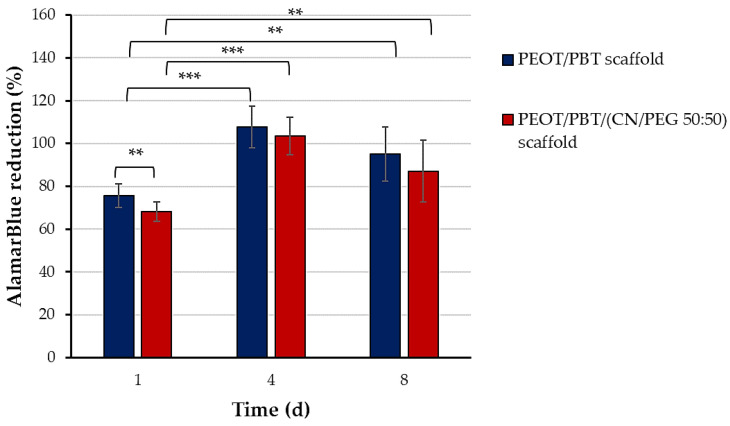
Bar graphs displaying the AlamarBlue^®^ assay results, indicated as dye reduction percent, of hMSCs differentiated into fibroblasts for 8 days on PEOT/PBT and PEOT/PBT/(CN/PEG 50:50) scaffolds. Data are expressed as mean ± standard deviation (*n* = 3; ** *p* < 0.01; *** *p* < 0.001).

**Figure 6:**
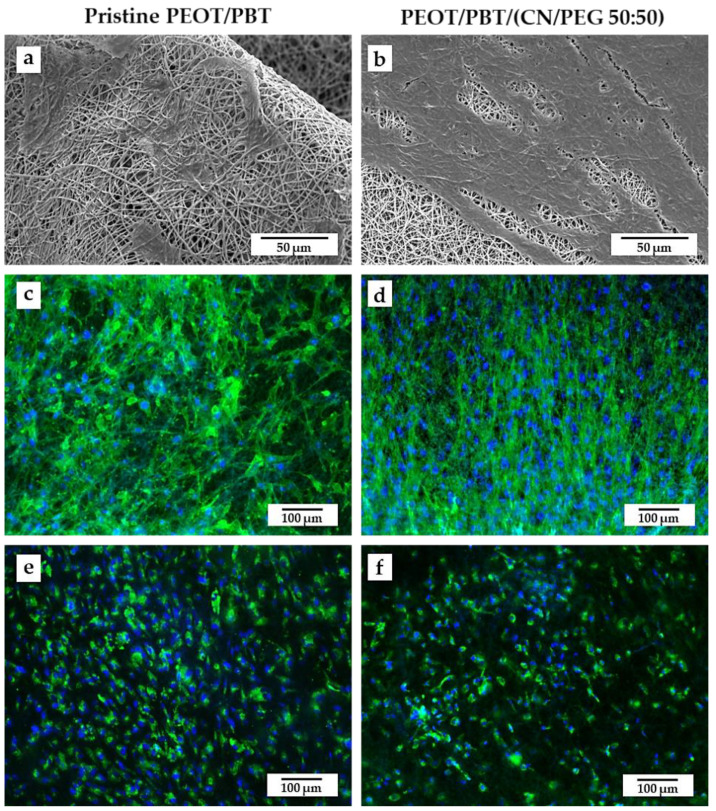
SEM micrographs of hMSCs differentiated into fibroblasts on (**a**,**c**,**e**) PEOT/PBT and (**b**,**d**,**f**) PEOT/PBT/(CN/PEG 50:50) scaffolds: (**a**,**b**) SEM micrographs imaging elongated cells adhered on the surface of the fiber meshes. Scale bar: 50 μm, high voltage (HV) 10 kV, magnification 1600×; (**c**,**d**) fluorescence micrographs revealing *f*-actin with phalloidin (green) and cell nuclei with DAPI (blue) staining; and fluorescence micrographs revealing collagen type I (green) immunofluorescence and DAPI (blue) staining. Scale bar: 100 μm, original magnification 100×.

The first step was to evaluate the capability of these scaffolds to be colonized by fibroblast-differentiated hMSCs. AlamarBlue^®^ test was performed to assess the metabolic activity of the cells cultured on PEOT/PBT and PEOT/PBT/(CN/PEG 50:50) scaffolds (Figure 5). At the endpoint, imaging analyses were performed to assess cell morphology and function (Figure 6).

On day 1, cell metabolic activity was significantly higher in PEOT/PBT than in PEOT/PBT/ (CN/PEG 50:50) scaffolds (*p* < 0.01); however, at later time-points, any statistically significant difference could not be detected between the pristine copolymer and the nanocomposite, thus confirming that the presence of CN/PEG (50:50) pre-composite did not affect the cell metabolism. A statistically significant increase in the metabolic activity over time was observed in both scaffold types between day 1 and day 8 (*p* < 0.01).

The morphology of fibroblast-differentiated hMSCs is reported in Figure 6a–d. Specifically, the cells were spread on the top of the meshes with an elongated morphology, as shown by SEM analysis (Figure 6a,b). In both the pristine copolymer and the nanocomposite scaffolds, *f*-actin was strongly expressed as a sign of good interaction with the substrates (Figure 6c,d). In addition, immunofluorescence detected the presence of collagen type I at intracellular level, as a sign of initial fibroblastic differentiation of hMSCs.

### 3.4. Anti-Inflammatory and Immune Response of HaCaT Cells

**Figure 7:**
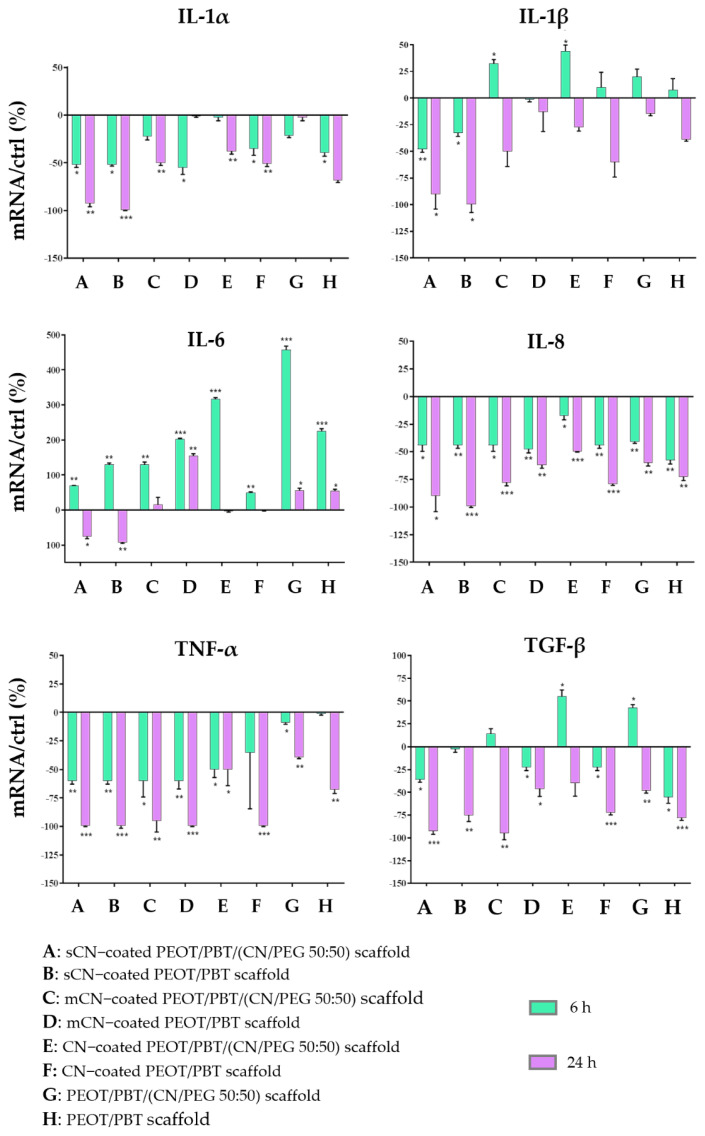
Bar graphs showing the results of real time RT-PCR related to different cytokines involved in the inflammatory response of HaCaT cells after being exposed for 6 h and 24 h to PEOT/PBT scaffolds, and PEOT/PBT/(CN/PEG 50:50) scaffolds, coated with electrosprayed CNs from different sources [namely, CNs (from crustaceans by Texol s.r.l.), sCNs (from shrimps, by Celabor s.c.r.l.), and mCNs (from mushroom by Celabor s.c.r.l.)]. The results were reported as percentage (%) normalized by the basal expression of those cytokines in untreated cells as control (ctrl). Data are expressed as mean ± standard deviation with respect to ctrl (*n* = 3; * *p* < 0.05; ** *p* < 0.01; *** *p* < 0.001).

Figure 7 shows the results of quantitative RT-PCR related to different cytokines involved in the inflammatory response of HaCaT cells after 6 h and 24 h of exposure to PEOT/PBT and PEOT/PBT/(CN/PEG 50:50) electrospun scaffolds coated with electrosprayed CNs from different sources, namely, crustacean (i.e., CNs), shrimp (i.e., sCNs) and mushroom (i.e., mCNs).

The reported outcomes showed that all the samples promoted anti-inflammatory activity in HaCaT cells, as they were able to strongly downregulate at 24 h, the expression of all the pro-inflammatory cytokines tested, except IL-6, which was definitely downregulated only by sCN coatings. After a general rise of this cytokine at 6 h, its expression was decreased again at 24 h in the samples. It has to be noted that the uncoated fibrous scaffolds were able to slightly change the expression of the pro-inflammatory cytokines studied. Overall, the most powerful downregulating effect of the coatings was played by sCNs.

Considering the anti-inflammatory marker, TGF-β, the nanocomposite scaffold also showed an early upregulation (at 6 h) of this cytokine, when it was coated with mCNs and CNs. The overall behavior of this molecular panel using different CNs, suggested an initial wound healing activity at early times, indicated by the rise of IL-6 in all the conditions and IL-1β in all but sCNs, followed by a marked resolution of the pro-inflammatory markers, including the powerful pro-inflammatory cytokine TNF-α with consequent reduction of the inflammatory state.

**Figure 8:**
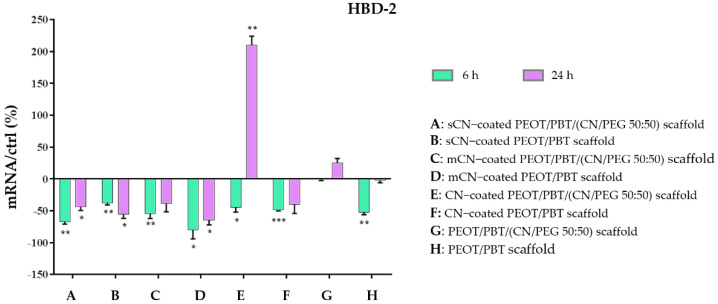
Bar graph showing the results of RT-PCR analysis for HBD-2 produced by HaCaT cells exposed for 6 h and 24 h to PEOT/PBT scaffolds, and PEOT/PBT/(CN/PEG 50:50) scaffolds, coated with electrosprayed CNs from different sources. The results were reported as percentage (%) normalized by the basal expression of those cytokines in untreated cells as control (ctrl). Data are expressed as mean ± standard deviation with respect to ctrl (*n* = 3; * *p* < 0.05; ** *p* < 0.01; *** *p* < 0.001).

We also investigated indirect antimicrobial activity in terms of mRNA expression of the HBD-2 at 6 h and 24 h. The outcomes, reported in Figure 8, showed that HBD-2 was slightly downregulated with respect to the basal conditions of HaCaT cells by all the treatments (range: 40–60%), but CN (from crustaceans) electrosprayed CN/PEG (50:50) nanocomposite scaffolds, which interestingly upregulated HBD-2 expression to 210% at 24 h. Additionally, the nanocomposite itself, produced using the same type of CNs was able to increase the HBD-2 gene expression by 30%. All in all, while the coatings with sCN and mCN negatively affected HBD-2 expression, CNs showed a significant indirect antimicrobial activity in combinations with PEOT/PBT/(CN/PEG 50:50) fiber scaffolds.

## 4. Discussion

Middle ear pathology is a leading cause of structural damage to the tissues involved in conductive hearing, which therefore need tissue substitutes [32,33]. Even though the otologic reconstructive surgery can avail itself of successful strategies to replace the damaged tissues and restore the acoustic function, the presence of an inflamed ear due to chronic infections with modified ventilation capacity supports pathology recurrence and perpetuation of inflammatory states, which overall challenge the stability of biomaterial-devices [2]. Indeed, non-biodegradable synthetic materials, also known as alloplastics, tend to be extruded, whereas biodegradable materials, including allografts, are quickly resorbed as a result of inflammatory and mechanical cues. As such, a tissue engineering approach based on long term biodegradable polymers, including poly(propylene fumarate)/poly(propylene fumarate−diacrylate) (PPF/PPF-DA) and PEOT/PBT has been proposed [6,10,34]. However, to preserve health and function of the middle ear after reconstructive surgery, the addition of features to control inflammation and possibly to fight recurrence of infections could be advantageous. Having replacements that can fulfill mechanical, regenerative, and immunomodulatory requirements could finally overcome the drawbacks of alloplastic materials and allografts in middle ear surgery.

Recently, CNs have attracted the interests of cosmetology and skincare for their green character, combined with excellent anti-inflammatory and antibacterial properties [13,15,29,30,35]. Since CNs can be biodegraded in the human body by resident enzymes, without risk of accumulating in tissues and organs, their use even in the biomedical field would be convenient, including the otologic devices. In this study, we investigated a strategy to enable CN availability in the middle ear for a long time, aimed at counteracting the inflammatory states and inducing a proper immune response. It is a fact that epithelial cells contribute to innate immunity by secreting factors involved in modulating inflammation and indirectly concur to antimicrobial actions [36]. Therefore, considering that the tympanic cavity is covered by epithelia and the eardrum itself is made up by outer squamous and inner mucosal epithelial layers [1], by controlling the response of these cells, we could modulate the tissue reactivity toward the newly implanted materials, thus favoring device acceptance and promoting the regenerative pathways in a natural wound healing manner. We combined two routes to make CNs available in the tissue environment across different timescales: *(i)* by producing a long term biodegradable nanocomposite made of CNs (as a filler) and PEOT/PBT (as a matrix), used to produce electrospun TM scaffolds; and *(ii)* by coating the surface of the fibrous scaffolds with CNs. In the first case, the CNs were considered to have a mechanical action as a part of the nanocomposite, an initially minimal biological action via surface exposure, along with a subsequently prolonged biological action upon copolymer biodegradation (e.g., in several month-time) and release of CNs from the bulk. In the second case, CNs were considered to be immediately available at the surface, so as to exert a biological activity in the short term after implantation. By this combined strategy, we could have CNs available for a prolonged time in the nearby tissues.

To have a mechanically reliable nanocomposites, a CN/PEG 50% (*w/w* %) pre-composite was chosen, having 2% CNs with respect to the copolymer weight (*w/w* %). This choice was derived from previous studies reporting on a viscosity mismatch between the two components in the melt, in particular using PLA [24,37,38]. In general, a number of CN/polymer nanocomposites, produced and processed from the molten state, have revealed improved mechanical properties along with anti-inflammatory and antimicrobial activity [24,39,40,41]. Some studies have reported on the feasibility of electrospinning CN/polymer nanocomposites, for example using CN/chitosan or CN/PCL solutions [42,43]. In fact, many parameters affect the electrospinning process [44], and the addition of CNs may vary the solution concentration, as well as the interaction with the electric field. Here, in line with other studies, which have widely demonstrated the electro-spinnability of pristine PEOT/PBT [6,8,10], we also showed that its nanocomposite PEOT/PBT/(CN/PEG 50:50) was successfully electrospun into ultrafine fibers. 

In order to test the capability of these fibers to withstand a long-term exposure within an inflamed tissue environment, the TM scaffolds were kept for 4 months in a degradation bath mimicking some features of the middle ear pathology. The otitis-simulating solution was prepared following typical concentration of salts, glucose, albumin, and lysozyme found in serous middle ear effusions (SMEE) of patients with COM with effusion, which is inflammation driven [45]. SMEE was chosen with respect to mucous effusion containing high levels of mucin, to avoid a high viscosity of the degradation bath, caused by the presence of mucin and its interaction with CNs, which would possibly result in a coating layer inhibiting diffusion phenomena [46,47]. Lysozyme is an antimicrobial enzyme present in exocrine secretions, which also concurs to the degradation of chitin. Although lysozyme concentrations in SMEE greatly vary in the literature, higher levels are always detected in children with respect to adult patients [45,46,48]. Considering all these differences, an average value of 2 mg/mL lysozyme concentration was chosen for this study. Due to the lightweight of real size TM scaffolds, any difference in weight could not be detected for the presence of precipitated organic molecules from the solution to the fibers. This phenomenon is also expected to occur in vivo. SEM analysis did not reveal any erosion phenomena on the fiber surface due to enzymatic attack to the CNs or other biodegradation events after 4 months, thus confirming the durability of the biomaterial in aggressive conditions and the capability of the copolymer to preserve CNs for even longer times.

The abovementioned fibrous meshes were thus electrosprayed using CNs from different sources, which have been previously tested for skincare applications using other fibrous materials as substrates [29,30]. PEOT/PBT-based fibers were found to be decorated by non-aggregated CNs. We analyzed the FTIR spectra of the different CNs, confirming their presence on the fibers. Interestingly, electrosprayed mCN showed an increased adhesion on the nanocomposite fibers compared to CNs extracted from fishing sources. This behavior can be attributed to the differences in chemical structure, physical-chemical properties, and processing methods used for extraction and purification of fungal versus fish chitins [49], and their effects on the electric field having the nanocomposite as a collecting substrate.

The next step was to assess the suitability, in particular of the nanocomposite, to act as a TM scaffold, thus allowing the eardrum connective tissue layer to be regenerated. Such a layer is made up by TM fibroblasts, which produce collagens (in particular type II, in addition to type I, III, and IV) and elastin [1,9]. Therefore, TM fibroblasts are different from dermal or lung fibroblasts, which synthesize predominantly collagen type I and elastin as the main proteins [50]. In a previous study, we explored the possibility of tailoring the collagen profiles in fibroblast-differentiating hMSCs adhered to electrospun scaffolds using a chondrogenic culture media, as it stimulates collagen type II expression [9]. In those experiments, we demonstrated that the application of mechanical forces, applied via a TM bioreactor, were necessary to have a proper hMSC commitment into TM-like fibroblasts, along with a chondrogenic medium and suitable mechanical properties of the scaffold. To pave the way for further investigations in dynamic culture conditions, in this study we used a chondro-differentiating medium to assess cell metabolic activity, therefore we analyzed *f*-actin expression and collagen type I synthesis. The obtained outcomes, revealed that cells were viable, well adhered to the fibers and produced collagen type I at an intracellular level after 8 differentiation days, without appreciable differences, at this stage of investigation, between PEOT/PBT and PEOT/PBT (CN/PEG 50:50) scaffolds. As previous studies have clearly shown the suitability of PEOT/PBT electrospun scaffolds for eardrum applications [6,8,10], we hypothesized that PEOT/PBT (CN/PEG 50:50) would contribute to improving the immunomodulatory properties of PEOT/PBT scaffolds, without compromising their regenerative capacity.

By interacting with HaCaT cells, chosen as a model of the epithelial layers of the TM, and specifically of the outer squamous epithelium [1], the pristine copolymer and the nanocomposite had a similar behavior towards the expression of pro-inflammatory markers. This is consistent with the low amount of CNs, i.e., 2% (*w/w* %), that was loaded in the copolymer. The fiber meshes were covered with different CN types, to exert a subitaneous contact with the keratinocytes, thus possibly inducing a quick anti-inflammatory response. All the samples enhanced an anti-inflammatory response in HaCaT cells, as they were able to strongly downregulate a panel of pro-inflammatory cytokines at 24 h. This is in line with previous findings reporting that the small diameter fiber scaffolds as obtained via electrospinning reduced the immune response of macrophages and keratinocytes [51,52]. In particular, only sCNs were able to downregulate the expression of IL-6, which is a powerful pro-inflammatory cytokine expressed in wounded keratinocytes [53]. In COM with effusions, high levels of IL-1α, IL-1β, and TNF-α have been reported [54]. In particular, IL-1β contributes to inflammatory cell infiltration into the middle ear, thus prolonging the diseased conditions. Additionally, in this case, sCNs were the most effective in downregulating this cytokine. In perforated acute otitis media (AOM) exudates containing live bacteria, IL-6 and IL-8 were also detected in high amounts [55]. IL-8 and TNF-α were the most markedly downregulated cytokines by all the samples tested. All these findings are supportive of the hypothesis that CNs can help to reduce inflammatory processes in the middle ear.

Finally, CN-coated PEOT/PBT/(CN/PEG 50:50) scaffold was the only one showing upregulation of the anti-inflammatory cytokine TGF-β, along with HBD-2, both of them being fundamental players in immunity [56,57]. In fact, by secreting antimicrobial peptides, such as HBD-2, epithelia protect the tissues from pathogens by an innate immune response, which is fundamental in tissue health, healing, and function [58]. Ultimately, this study showed that CNs from crustaceans exerted a more powerful action than the mCNs, among our samples. Since preparation procedures and source lots may strongly influence the outcomes, a systematic investigation is necessary to understand in-depth the correlation between structure, processing and function of CNs to develop efficient natural-based bioactive replacements. To this purpose, computational modeling approaches show a high relevance [59]. Having robust processes to produce bio-based fully biocompatible nanofibrils able to modulate inflammatory and immune response could greatly contribute to developing tissue replacements able to improve the quality of life of patients and reduce healthcare costs and drug consumption.

## 5. Conclusions

We investigated in vitro a putative role of CNs as anti-inflammatory agents to be employed in middle ear replacements. Specifically, an electrospun nanocomposite TM scaffold incorporating CNs was set-up using PEOT/PBT as a copolymer matrix, as it has a long biodegradation time. It was shown that, in an otitis-simulating fluid, that the TM scaffolds were stable up to 4 months. In addition, preliminary tests performed using fibroblast-differentiated hMSCs demonstrated that the nanocomposite was comparable to the widely tested pristine copolymer in terms of cell viability, adhesion, and collagen type I expression. The fibrous meshes were further coated with CNs from different sources, namely crustaceans (i.e., CNs), shrimp (i.e., sCNs), and mushrooms (i.e., mCNs), via electrospray, and a panel of cytokines involved in the inflammatory and immune response was tested. The obtained findings demonstrated that the surface functionalization with nanochitins was effective in downregulating the pro-inflammatory cytokines assayed, which are greatly involved in COM with effusions and also AOM exudates. For most of them, it was shown that CNs from crustaceans could strengthen the innate immune response of HaCaT keratinocytes, also by markedly upregulating the antimicrobial peptide HDB-2 and the anti-inflammatory cytokine TGF-β, thus appearing as a valid option for developing novel TM scaffolds.

## Figures and Tables

**Table 1:** Operating parameters for electrospinning PEOT/PBT and PEOT/PBT/(CN/PEG 50:50) (T = temperature, H = humidity).

Operating Parameters	PEOT/PBT/(CN/PEG 50:50)
Concentration	18% (*w/v*) in CHCl_3_:HFIP = 70:30
Collector type, rotating speed	Rotating drum covered with PP sheet ^1^, 150 rpm
Needle diameter	800 μm
Ambient parameters	T = 23 °C and H = 40%
Voltage applied	20 kV (grounded collector)
Flow rate	900 μL/h
Tip to collector distance	10 cm

^1^ Collector used for the optimized electrospinning of PEOT/PBT/(CN/PEG 50:50), PP holes consistent with the TM size.

**Table 2:** Analyzed genes and RT-PCR conditions to assess the inflammatory and immune response of HaCaT cells exposed to the different CN-treated PEOT/PBT and PEOT/PBT/(CN/PEG) scaffolds.

Gene	Primer Sequence (Forward and Reverse)	Conditions	Base Pairs
IL−1α	5′−CATGTCAAATTTCACTGCTTCATCC−3′ 5′−GTCTCTGAATCAGAAATCCTTCTATC−3′	5 s at 95 °C, 8 s at 55 °C, 1 s at 72 °C for 45 cycles	421
IL−1β	5′−GCATCCAGCTACGAATCTCC−3′ 5′−CCACATTCAGCACAGGACTC−3′	5 s at 95 °C, 14 s at 58 °C, 28 s at 72 °C for 40 cycles	708
TNF−α	5′−CAGAGGGAAGAGTTCCCCAG−3′ 5′−CCTTGGTCTGGTAGGAGACG−3′	5 s at 95 °C, 6 s at 57 °C, 13 s at 72 °C for 40 cycles	324
IL−6	5′−ATGAACTCCTTCTCCACAAGCGC−3′ 5′−GAAGAGCCCTCAGGCTGGACTG−3′	5 s at 95 °C, 13 s at 56 °C, 25 s at 72 °C for 40 cycles	628
IL−8	5−ATGACTTCCAAGCTGGCCGTG−3′ 5−TGAATTCTCAGCCCTCTTCAAAAACTTCTC−3′	5 s at 94 °C, 6 s at 55 °C, 12 s at 72 °C for 40 cycles	297
TGF−β	5′−CCGACTACTACGCCAAGGAGGTCAC−3′ 5′−AGGCCGGTTCATGCCATGAATGGTG−3′	5 s at 94 °C, 9 s at 60 °C, 18 s at 72 °C for 40 cycles	439
HBD−2	5′−GGATCCATGGGTATAGGCGATCCTGTTA−3′ 5′−AAGCTTCTCTGATGAGGGAGCCCTTTCT−3′	5 s at 94 °C, 6 s at 63 °C, 10 s at 72 °C for 50 cycles	198

## Data Availability

Data are available upon request to the corresponding author.
